# Identification of Single Nucleotide Polymorphisms Associated with Brown Rust Resistance, α-Amylase Activity and Pre-harvest Sprouting in Rye (*Secale cereale* L.)

**DOI:** 10.1007/s11105-017-1030-6

**Published:** 2017-04-26

**Authors:** Monika Rakoczy-Trojanowska, Paweł Krajewski, Jan Bocianowski, Małgorzata Schollenberger, Wojciech Wakuliński, Paweł Milczarski, Piotr Masojć, Małgorzata Targońska-Karasek, Zofia Banaszak, Katarzyna Banaszak, Waldemar Brukwiński, Wacław Orczyk, Andrzej Kilian

**Affiliations:** 1 Warsaw University of Life Sciences, Warsaw, Poland; 2 Polish Academy of Sciences Botanical Garden – Centre For Biological Diversity Conservation, Powsin, Warsaw, Poland; 3 Institute of Plant Genetics, Polish Academy of Sciences, Poznań, Poland; 4 Poznań University of Life Sciences, Poznań, Poland; 5 West Pomeranian University of Technology, Szczecin, Poland; 6 Danko Plant Breeders LTD, Kościan, Poland; 7 The Plant Breeding and Acclimatization Institute – National Research Institute, Radzików, Poland; 8 Diversity Arrays Technology P/L, Canberra, Australia

**Keywords:** Genome-wide association mapping, Candidate gene association mapping, *ScBx* genes, DArTSeq, Kompetitive allele specific PCR

## Abstract

Rye is a crop with relatively high resistance to biotic and abiotic stresses. However, the resistance to brown rust (*Puccinia recondita* f. sp. *secalis*) and pre-harvest sprouting are still not satisfactory. High α-amylase activity is also among the main disadvantages of this species. Therefore, effective tools, e.g. molecular markers, allowing precise and environmentally independent selection of favourable alleles are desirable. In the present study, two kinds of association mapping—genome-wide association mapping (GWAM) based on sequences of DArTSeq markers and candidate gene association mapping (CGAM) based on sequences of *ScBx* genes—were chosen for development of molecular markers fulfilling these criteria. The analysed population consisted of 149 diverse inbred lines (DILs). Altogether, 67 and 11 single nucleotide polymorphisms (SNPs) identified in, respectively, GWAM and CGAM, were significantly associated with the investigated traits: 2 SNPs with resistance to brown rust, 71 SNPs with resistance to pre-harvest sprouting and 5 SNPs with α-amylase activity in the grain. Fifteen SNPs were stable across all environments. The highest number (13) of environmentally stable SNPs was associated with pre-harvest sprouting resistance. The test employing the Kompetitive Allele Specific PCR method proved the versatility of four markers identified in both GWAM and CGAM.

**Electronic supplementary material:**

The online version of this article (doi:10.1007/s11105-017-1030-6) contains supplementary material, which is available to authorized users.

## Introduction

Rye (*Secale cereale* L.) is one of the most important cereals in Eastern, Central and Northern European countries. It was grown on approximately 5.8 million ha worldwide in 2013 [http://faostat.fao.org]. Its importance results both from the unique properties such as low water and soil fertility requirements and good tolerance for biotic and abiotic stresses, good overwintering, high nutritional value connected with the content of dietary fibre and the multipurpose usage—for bread making, animal feed and alcohol production. Recently, it is also gaining attention as a biomass crop (Bushuk 2001). In spite of many advantages, rye exhibits some weaknesses negatively affecting yield quality and quantity, among which the most important are the relatively low resistance to brown rust (*Puccinia recondita* f. sp. *secalis*), pre-harvest sprouting (PHS) and high α-amylase activity.

*Puccinia recondita* f. sp. *secalis*, an obligate biotrophic basidiomycete fungus, is a commonly occurring and widely distributed major rye pathogen (Roux and Wehling 2010). A rust epidemic results in premature leaf senescence and in consequence a reduced number of florets in the spike. The disease intensity is correlated with reduction of kernel number, yield quality and quantity. Available estimates indicated 11 to 27% thousand kernel weight reduction (Miedaner and Sperling 1995) and yield decrease of up to 80% (Solodukhina 1997 2002) depending on genotype. Modern rye varieties do not always exhibit a satisfactory level of resistance to brown rust (Solodukhina 1997; Miedaner et al. 2002; Roux et al. 2004). This problem, in particular, concerns the population varieties which dominate in Poland. Wehling et al. (2003) identified in rye two dominant genes, *Pr1* and *Pr2*, both conferring resistance through hypersensitivity, which were recognized as active against a local population of *P. recondita* as well as isolates of the fungus obtained from single pustules and representing various virulence. Three additional genes, *Pr3*, *Pr4*, *Pr5*, associated with race-specific resistance to brown rust, were identified by Roux et al. (2004). Specific resistance is an effective strategy, often used in breeding, against *P. recondita* as well as other biotrophic pathogens, but unfortunately its durability is not satisfactory (Miedaner et al. 2012). An alternate approach focuses on nonspecific resistance. It is also known as non-isolate-specific or partial resistance as the disease development is reduced but not completely stopped.

So far, nonspecific resistance genes coding for ABC transporter and proline-containing protein have been recognized in wheat (Krattinger et al. 2009) and in rice (Fukuoka et al. 2009). The concept of nonspecific resistance against brown rust of rye has not been studied in depth.

PHS is the phenomenon occurring during the later stages of cereal grain development when prolonged rain induces premature germination whilst the grain is still in the ear. Water absorbed by the rye kernel activates enzymes, especially α-amylase, which causes degradation of the endosperm starch and a significant reduction of grain quality. Germinated seeds represent material of low sowing and technological value. PHS has been considered to be one of the main factors negatively affecting the yield and quality of crops grown in wet or humid weather conditions. PHS resistance is a complex quantitative trait, controlled by a number of quantitative trait loci (QTL) mapped on each of the seven rye chromosomes (Masojć and Milczarski 2009; Masojć et al. 2009; Myśków et al. 2012). Studies of QTL controlling pre-harvest sprouting and α-amylase activity in rye demonstrated that these two complex genetic systems coincide to a great extent (Masojć and Milczarski 2009; Myśków et al. 2012).

Increasing resistance to rust, PHS and lowering of α-amylase activity are still current objectives in breeding of rye varieties. Finding molecular markers for these agronomic traits is therefore essential, since application of novel molecular tools in breeding will speed up selection of valuable genotypes.

Association mapping, also called linkage disequilibrium mapping, involves searching for genotype (usually individual single nucleotide polymorphisms (SNPs) or SNP haplotypes)—phenotype correlations in unrelated individuals using dedicated statistical methods (Abdurakhmonov and Abdukarimov 2008; Zhu et al. 2008; Rafalski 2010). The association mapping approach creates possibilities to generate good quality markers for marker-assisted selection (MAS). The most effective are functional markers reflecting gene polymorphism tightly linked with the trait which is a direct cause of phenotypic variation. Association mapping provides opportunities to find such markers in a broad spectrum of genetic resources. Its potential results from the likelihood of higher mapping resolution because of the utilization of more recombination events in the germplasm’s developmental history (Abdurakhmonov and Abdukarimov 2008; Rafalski 2010). Thus, association mapping has become a promising approach compared to traditional linkage mapping. There are two main types of association mapping: genome-wide association mapping (GWAM) and candidate gene association mapping (CGAM). The GWAM approach surveys genetic variation in the whole genome to find signals of association for various complex traits, whereas CGAM correlates DNA polymorphisms in selected candidate genes and the trait of interest (Zhu et al. 2008; Rafalski 2010).

In our research, DArTSeq markers were used for GWAM and *ScBx* genes for CGAM. DArTseq technology utilizes the DArT marker platform in combination with next-generation sequencing platforms and represents a new implementation of sequencing of complexity-reduced representations (Sansaloni et al. 2011; Courtois et al. 2013). *ScBx1–5* genes, recently sequenced and characterized by Bakera et al. (2015), control the five steps of benzoxazinoid (BX) biosynthesis (starting with the conversion of indole-3-glycerol phosphate to indole followed by four monooxidations with the final product 2,4-dihydroxy-1,4-benzoxazin-3-one). BXs are protective and allelopathic secondary metabolites are found in a large number of species belonging to the *Poaceae* family, including the major agricultural cereals maize, wheat and rye (Frey et al. 2009; Niemeyer 2009). Several studies have shown a relationship between BX and disease resistance (Niemeyer 2009). BXs have also been shown to play a role in the inhibition of gibberellin-induced α-amylase activity in barley seeds (Kato-Noguchi 2008). Thus, genes encoding enzymes participating in BX biosynthesis related enzymes seem to be good candidates for association mapping.

In cereals, mainly in wheat, there are many examples of successful application of association analysis for mapping QTL controlling PHS resistance (Lohwasser et al. 2013; Albrecht et al. 2015), rust resistance (Philomin et al. 2015; Kertho et al. 2015), α-amylase activity (Emebiri et al. 2010) or frost tolerance (Li et al. 2011).

So far, a relatively large number of molecular markers have been developed for rye: RAPD (Masojć et al. 2001; Bednarek et al. 2003), RFLP (Korzun et al. 2001; Masojć et al. 2001), AFLP (Bednarek et al. 2003), SSR (Saal and Wricke 1999; Khlestkina et al. 2004), DarT (Bolibok-Brągoszewska et al. 2009; Milczarski et al. 2011), GBS (Milczarski et al. 2016), and there are many examples of their successful use for tagging certain traits, e.g. PHS (Masojć and Milczarski 2009; Masojć et al. 2009; Myśków et al. 2012), aluminium tolerance (Gallego et al. 1998) or frost tolerance (Li et al. 2011). Nevertheless, no attempts have been made to perform association analysis of PHS, rust resistance and α-amylase activity nor have DArTSeq markers been used for such studies in rye yet.

The aims of the present study were the following: (1) to characterize a broad collection of rye diverse inbred lines representing the variability among Polish breeding materials with respect to brown rust resistance, pre-harvest sprouting and α-amylase activity, (2) to identify SNPs in *ScBx* genes and DArTSeq markers associated with these traits.

## Materials and Methods

### Plant Material

The plant material used in the experiments consisted of 153 diverse inbred lines (DILs) bred in two Polish breeding companies—Danko Plant Breeders Ltd. (40 DILs, designated as D1–D40) and Poznań Plant Breeders Ltd. (30 lines, designated as P1–P30); Botanical Garden of Polish Academy of Science (8 lines; OG1–OG8); Warsaw University of Life Sciences, Department of Plant Genetics, Breeding and Biotechnology (5 lines designated as W1–W5); West Pomeranian University of Technology, Department of Plant Genetics, Breeding and Biotechnology (20 lines designated as S1–S20); and Wrocław University of Environmental and Life Sciences, Department of Genetics, Plant Breeding and Seed Production (50 lines designated as WR1–WR50).

In 2013, 103 lines were tested, and the set was increased to 153 lines in 2014.

The name W1 is a synonym of L318. Additional plant material, not related (but belonging to the same Petkus germplasm pool) to DILs used in the association analysis, consisting of 60 recombinant inbred lines (RIL) from the mapping population 541 × Ot1-3 (Milczarski et al. 2007) and 20 inbred lines from Danko Plant Breeders Ltd. was used in the Kompetitive allele-specific PCR (KASP) analysis. The inbreeding degree of DILs was at least S5 (usually higher), except for WR lines, for which it was S4.

### Methods

#### Phenotyping

Each line was represented by 33 plants per location and year, grown in a randomized block design (11 plants per replicate). The experiments were performed in two seasons, 2013 and 2014, in two locations: an experimental field of the West Pomeranian University of Technology in Szczecin (N 53° 26′ 51.291″, E 14° 31′ 42.1278″) and Danko Plant Breeders Ltd. in Choryń (N 52° 02′ 19.7″, E 16° 46′ 09.0″). Together, observations from four experiments designated as WPUT 2013, WPUT 2014, DANKO 2013 and DANKO 2014 were scored.

The following traits of DILs were phenotyped: brown rust resistance (R-R), pre-harvest sprouting resistance (PHS-R) and α-amylase activity in grain (AMY).

##### R-R

The reaction of DILs to *P. recondita* was examined in field conditions where the source of inoculum was uredospores of a local population of the fungus, naturally occurring in the environment and spontaneously infecting the plants. No special treatments were applied for enhancing/increasing the infection potential of the fungus. For comparison purposes, the set of tested lines was supplemented with two rye cultivars, Dańkowskie Złote as a susceptibility reference and Bosmo as a tolerance reference.

Rating was performed at maximum brown rust epidemic intensity and based on average evaluation of all plants in the plot.

Plant infection level was evaluated according to a six-degree scale:

- 0—resistant, no symptoms on plants
- 1—resistant, chlorosis/necrosis and/or single uredinia sporadically visible on leaves
- 2—moderate resistant; single small uredinia regularly present on majority of plants
- 3—moderately susceptible; up to 40% of leaf surface covered with uredinia
- 4—susceptible; up to 70% of leaf surface covered with uredinia
- 5—very susceptible; over 70% of leaf surface covered with uredinia.

##### PHS-R

Three to six spikes from each DIL and each replicate were harvested at full ripeness and kept at room temperature for 3 days. Then, the spikes were sprayed with water for 5 min each day and kept in a moisture chamber at 20 °C for a period of 7 days. Resistance to PHS was determined as a percentage of germinated kernels (the lower the percentage, the higher the resistance).

##### AMY

1.5 g of kernels with no visible signs of sprouting from each DIL and each replicate was milled into flour. Grain used for the α-amylase assay was stored at room temperature for the first 2 weeks after harvest and then kept at 4 °C for the few months before analysis. For α-amylase extraction, 1 g of flour was mixed with 4 ml of distilled water, equilibrated for 10 min at room temperature and finally centrifuged at 8000 rpm at 4 °C for 20 min. Alpha-amylase activity in mature grain was detected using a simple gel diffusion method (Masojć and Larsson-Raźnikiewicz 1991) where the diameter of the diffusion circle shows a linear relationship with the logarithm of the enzyme activity given in units per milliliter. A calibration curve was obtained by a series of dilutions of a commercial alpha-amylase from barley malt (Sigma).

The number of lines phenotyped in terms of PHS-R and AMY was slightly lower than the number of lines evaluated in respect of R-R in some experiments (Table 1) because of plant losses before the spike harvest began. In two trials (DANKO 2013 and WPUT 2014), one and three RILs, correspondingly, had delayed vegetation, and because of this, they were not evaluated in terms of R-R.

**Table 1:** Statistical characteristics of observations in field experiments

Experiment	Number of DILs	Mean	Std. error of mean value	Minimum	Maximum	Coefficient of variation
R-R (0–5 scale)						
DANKO 2013	102	3.56	0.07	0.67	5.00	19.93
DANKO 2014	152	3.66	0.05	1.00	5.00	16.08
WPUT 2013	103	3.38	0.06	1.50	4.67	19.02
WPUT 2014	149	2.97	0.06	0.00	4.00	23.65
PHS-R (%)						
DANKO 2013	100	37.12	1.51	8.63	78.42	40.59
DANKO 2014	145	36.50	1.38	4.08	84.19	45.51
WPUT 2013	103	44.30	3.11	3.54	98.75	71.22
WPUT 2014	150	53.97	2.11	8.00	95.81	47.86
AMY (U/mL)						
DANKO 2013	98	11.97	0.10	9.00	16.00	8.67
DANKO 2014	151	9.43	0.07	6.67	13.38	9.04
WPUT 2013	102	10.35	0.08	8.50	14.75	8.24
WPUT 2014	148	10.90	0.09	7.50	15.25	10.47

#### Genotyping and SNP Data Processing

Genotypic data for association mapping came from polymorphisms identified in DArT and candidate gene sequences.

##### DArT Sequences

One hundred forty-eight DILs were genotyped. The preparation for DArTSeq genotyping was performed as described by Bolibok-Brągoszewska et al. (2009). Briefly, DNA was isolated from bulked (eight plants per each DIL), lyophilised two-week-old leaves using the CTAB method (Murray and Thompson 1980). DNA purity and concentration were evaluated using a NanoDrop 2000 spectrophotometer (Thermo Scientific, Waltham, USA). DArTseq genotyping was done in the Diversity Arrays Technology Pty. Ltd., Australia, using the platform developed by Cruz et al. (2013). SNPs were extracted with the accompanying metadata (including average marker reproducibility calculated from technical replications). For the association analysis, only DArT sequences meeting the following criteria were selected: one SNP within a given sequence (69 nt), minor allele frequency (MAF) > 0.25 and the missing observation fractions <10%. SNP sequences were mapped in Sce assembly 02 (Haseneyer et al. 2011) using the Blast service at https://www.gabipd.org/ with default parameters.

##### *ScBx* Genes

*ScBx1-5* genes (comprising exons, introns, 3′UTRs and promoters, except for *ScBx1* in which no 3′UTRs were assayed; Table SI, Electronic Supplementary Material, ESM), previously sequenced and characterized in rye inbred line L318 (Bakera et al. 2015), were re-sequenced in a set of 143 DILs using the Roche 454 sequencing method (Genomed S.A., Warsaw). The smaller number of DILs included in the analysis than the total number of phenotyped lines resulted from the inability to obtain the common fragment of the 3′UTR of the *ScBx5* gene, in five lines. The Roche 454 paired end reads (median read length = 190 nt, median number of reads per DIL = 320,587, min = 900, max = 7,211,998) were mapped to the reference sequence using Bowtie 2 (Langmead et al. 2009), allowing for a maximum of two mismatches. The mapping results were processed by the SNP calling pipeline based on Samtools (mpileup) and Bcftools (index, call) software (Li et al. 2009; Li 2011, software online documentation).

The alleles the same as present in the reference line L318 were marked as REF, and the alleles resulting from a given SNP were marked as ALT.

##### Statistical Analysis and Association Mapping

Data were analysed by ANOVA in a model with fixed effects of years (Y), locations (L), and of Y × L interaction, and random effects of DILs and DIL × (Y, L) interactions. Association mapping, based on SNP data and on mean values of traits obtained in individual experiments, was done using the method allowing for interaction of genetic effects with the environment developed by van Eeuwijk et al. (2010) and Malosetti et al. (2013), based on the mixed linear model with the population structure estimated by eigenanalysis (principal component analysis applied to all markers). The compound symmetry variance-covariance model was used for environmental variation. All analyses and visualizations of results were done in GenStat 17 (EVSN Int. 2013). Significance of associations between traits and DArTseq markers was assessed on the basis of *P* values corrected for multiple testing by the Benjamini-Hochberg method as implemented in R function p.adjust.

#### Mapping DArTSeq Markers

The chromosome position of DArTSeq markers significantly associated with investigated traits at *P* < 0.001 was analysed based on a genetic map of population S (Milczarski et al. 2011) which was extended with DArTseq markers (Milczarski et al. 2016). This S map was a reference. For the SNPs, which could not be localized on the S map, other genetic maps were used: a genetic map based on population H (Milczarski et al. 2011) extended with DArTseq markers (Bolibok-Brągoszewska, personal communication) and a de novo constructed map based on population 541 × 723 comprising DArTseq markers (Milczarski et al. personal communication). The positions of SNPs (verified through ID number and sequence) on these maps were recalculated in relation to the reference map. For the estimation of position, SNP shared markers from compared maps were used.

For the construction of genetic maps, the program JoinMap 4.0 with the ML (maximum likelihood) mapping algorithm was applied.

#### Evaluation of Associations

Selected SNPs found to be significantly and stably associated with R-R and PHS-R were evaluated using the KASP assays (LGC Genomics, Herts, England). An additional selection criterion was the ability to design good quality primers for the analysed sequence. The 50 bp sequences, each upstream and downstream around the SNP, were the templates for design of primer pairs. KASP reactions were run in 10 μl reactions including 5 μl of KASP master mix, 0.14 μl of primer mix (LGC Genomics, Herts, England), and 5 μl of 17–20 ng/μl genomic DNA. PCR and fluorescent endpoint readings were carried out using the CFX96 Touch Real-Time PCR Detection System (Bio-Rad). Altogether, 120 lines were genotyped: 40 DILs included in the association analysis (experimental set, EXS)—20 DILs in the experiment on SNPs associated with PHS-R and 20 DILs in the experiment on SNPs associated with R-R—and 80 lines not included in the association analysis (evaluation set—EVS), comprising 60 recombinant inbred lines (40 taken for the experiment on SNPs associated with PHS-R and 20 for those associated with R-R) and 20 inbred lines (taken for the experiment on SNPs associated with R-R) phenotyped previously by Masojć et al. (2009) and by breeders from the Danko Plant Breeders Ltd., respectively. The lines for KASP test were chosen randomly from extreme groups of phenotypes. Each subset was composed in half of resistant and in half of susceptible lines, but the phenotypes were not revealed until completing KASP analysis.

## Results

### Phenotyping

The comparison of R-R, PHS-R and AMY levels in all experiments is shown in Table 1. The highest resistance to brown rust was observed in WPUT 2014, whereas the lowest R-R value was observed in DANKO 2014. Oppositely, for PHS, the highest resistance was observed in DANKO 2014 and the lowest one in WPUT 2014. The lowest activity of AMY was also noted in the DANKO 2014 trial whilst the highest AMY enzymatic activity was noted in the same location but in 2013. Results of ANOVA (Table SII, ESM) demonstrated that for all traits, the differences between years, locations and the year × location interaction were significant. Comparison of variance components among themselves and with their standard errors showed that the component for DIL genotypes dominates the interaction component for R-R, whereas for PHS-R, the component for interaction of DIL genotypes with locations was the largest.

Correlations of observed trait levels were not significant (Table 2) except for AMY and R-R, which were correlated in one experiment—WPUT 2013. The interactions of lines with years were larger—in relation to their standard errors—than the interactions with locations. The relationships between variance components are reflected in the estimates of broad-sense heritability, with the largest value for R-R (Table SII, ESM). Phenotypic and genetic correlations between environments (Table 3) confirm a stronger relationship between locations than between years for R-R and PHS-R, and an outlying behaviour of observations in WPUT 2013 for AMY.

**Table 2:** Correlations of observed mean values for DILs in four experiments

Experiment	Trait	R-R	PHS-R	AMY
DANKO 2014	R-R	1		
	PHS-R	−0.048	1	
	AMY	0.070	0.046	1
DANKO 2014	R-R	1		
	PHS-R	0.101	1	
	AMY	0.005	0.131	1
WPUT 2013	R-R	1		
	PHS-R	0.107	1	
	AMY	0.169*	−0.020	1
WPUT 2014	R-R	1		
	PHS-R	−0.003	1	
	AMY	0.061	−0.002	1

*Significant at *P* < 0.05

**Table 3:** Phenotypic and genetic correlations between environments for observed traits

Correlation between	In	R-R		PHS-R		AMY	
		Phenotypic^a^	Genetic^b^	Phenotypic^a^	Genetic^b^	Phenotypic^a^	Genetic^b^
2013–2014	DANKO	0.64	0.84	0.32	0.44	0.40	1.00
	WPUT	0.57	0.79	0.39	0.56	*0.14*	0.51
DANKO - WPUT	2013	0.65	0.86	0.31	0.66	*0.00*	0.48
	2014	0.74	0.96	0.62	0.69	0.55	1.00

^a^Correlations given in italics are not significant at *P* < 0.01

^b^Estimated in the mixed linear model with (year x location) combinations treated as four environments with fixed effects, and with unstructured genetic covariance matrix for interaction of lines and environments

The groups of lines provided by different breeders differed with respect to mean R-R and AMY, whereas for PHS-R, the differences were not significant (Table 4). Lines D had the significantly lowest mean level of R-R, and lines S had the significantly highest level of AMY.

**Table 4:** Comparison of groups of lines

Group of lines	R-R	PHS-R	AMY
D	3.136^a^	41.35^a^	10.60^a^
OG	3.505^bc^	51.01^a^	10.70^a^
P	3.393^b^	43.89^a^	10.41^a^
S	3.743^c^	42.81^a^	11.24^b^
W	3.509^bc^	52.33^ab^	10.25^a^
WR	3.461^b^	40.12^a^	10.61^a^
*P* value for difference between groups	<0.001	0.055	<0.001
LSD_0.05_	0.29	10.65	0.56

### Genotyping Data and Population Structure

#### DArTSeq

**Fig. 1:**
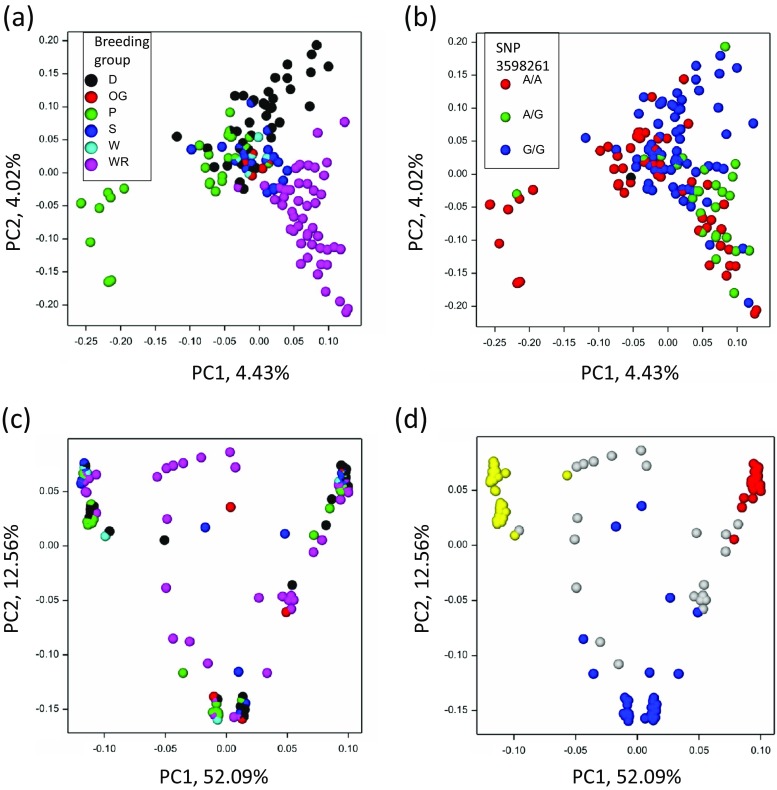
Population structure estimated by eigenanalysis

Out of 36,894 polymorphisms obtained, 2330 SNPs were selected for GWAM using the criteria specified above. Population structure estimated by eigenanalysis (Fig. 1a) shows large subsets of D and P lines (provided by breeding companies) differentiated from the others. The rest of DILs from these two groups were similar to OG, S and W lines, and form the central cluster in the graph. The WR lines were also separated from the others; their homozygosity (median 0.75; from 0.51 to 0.90) was lower than that of the rest of the lines (median 0.98; from 0.62 to 0.99). By performing the χ (Albrecht et al. 2015) independence test for origin of lines and genotypic classes for SNPs (details not shown), SNP 3598261 was identified as explaining well the dispersion of lines D (mostly G/G) and P (mostly A/A) (Fig. 1b). The fraction of homozygous observations for individual SNPs was from 0.53 to 0.97.

#### *ScBx* Genes

**Fig. 2:**
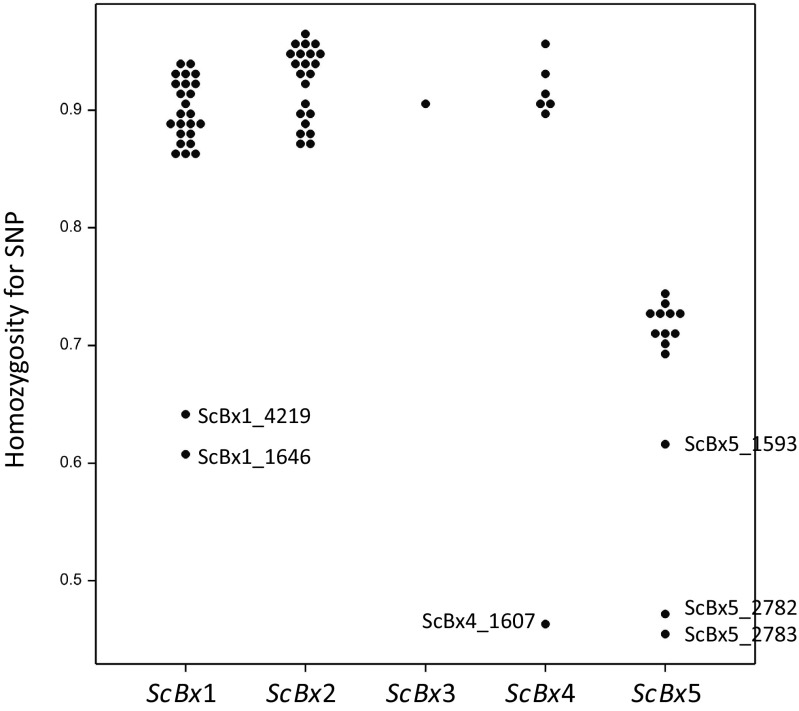
Homozygosity for SNP

In the sequences comprising full *ScBx* genes and their promoters, 70 SNPs were identified in a set of 143 DILs—26, 22, 1, 7 and 14 in *ScBx1*, *ScBx2*, *ScBx3*, *ScBx4* and *ScBx5* genes, respectively. All 70 polymorphisms were included in CGAM. Homozygosity of DIL with respect to these SNPs ranged from 0.28 to 1.00. There were six SNPs with the fraction of homozygous observations less than 0.65 (Fig. 2). In general, the SNPs located in *ScBx5* were mostly heterozygous. The population structure estimated by eigenanalysis (Fig. 1c) shows three separate groups of lines; however, the dispersion of lines does not exhibit any relationship to breeding groups. The population structure was determined mostly by the genotypes observed in genes *ScBx1* and *ScBx2*, for example, by a combined genotype at SNPs ScBx1_4491 and ScBx2_212 (Fig. 1d).

### Association Mapping: CGAM, GWAM

#### GWAM

The Q-Q plots of estimated allelic effects for all SNPs (Fig. S1, ESM) show their approximate normality and relatively large size of the extreme effects, especially for PHS-R. Based on the mixed linear model analysis, the number of SNPs with associations with investigated traits significant at FDR < 0.05 in GWAM was 67 (Table SIII, ESM). For R-R, one significant association was found (marker 3363612) with a negative (favourable) effect of the alternative allele. For PHS-R, 61 associations were detected (7 stable across all environments). Four associations that were stable over environments had a negative (favourable) effect of the alternative allele. For AMY, there were five significant associations, one with positive effects stable over environments. For PHS-R, for the associations with an interaction, the smallest allelic substitution effects were observed in almost all cases in WPUT 2013, and the largest in DANKO 2013; the mean allelic effect over environments was negative (favourable) in 35 cases and positive (unfavourable) in 23. All SNPs in DArT sequences significantly and stably associated with the traits of interest are listed in Table 5.

**Table 5:** SNPs in DArT sequences and *ScBx* genes significantly and stably associated with R-R, PHS-R and AMY

Type of association analysis	SNP ID/(consensus map position—chromosome position)^a^	SNP location	R-R		PHS-R		AMY	
			−log_10_ (*P* value)^a^	Allelic effect^b^	−log_10_ (*P* value)	Allelic effect ^b^	−log_10_ (*P* value)^a^	Allelic effect ^b^
GWAM	5200240/-	X			2.94	3.88		
	5217029/5RL, 160^Mil^	X			2.97	2.81		
	3745935/6RS, 35.9 ^Mil^	X			3.46	−4.72		
	5802575/3RL, 105 ^Mil^	X			3.23	−3.16		
	3595498/6RS, 15.9 ^Mil^	X			3.45	−3.94		
	3602388/2R, 110.7^Mar^	X			3.35	−2.33		
	7104304/-				2.94	1.06		
	3581291/1RL, 168.7 ^Mil^	X					4.02	0.24
CGAM	ScBx4_1583/5RS	First intron	2.24	−0.14				
	ScBx1_1367^c^/7RS	Promoter			2.35	2.82		
	ScBx1_2474/7RS	Promoter			2.08	2.68		
	ScBx1_4515^c^ /7RS	Seventh exon			2.19	2.61		
	ScBx1_4663/7RS	3′UTR			2.53	2.70		
	ScBx1_4736/7RS	3′UTR			2.20	2.36		
	ScBx4_1627/5RS	First intron			2.17	2.97		

Map positions of *ScBx* genes—according to Sue et al. (2011). ^Mil^—maps of Milczarski et al. (2011); Milczarski et al. (2016); Bolibok-Brągoszewska (unpublished) and Milczarski et al. (unpublished); ^Mar^—map of Martis et al. (2013)

^a^For GWAM-based markers

^b^Allelic (SNP) effects refer to the ALT allele with respect to the REF allele

^c^Markers validated in KASP analysis;

Out of 67 SNPs significantly associated with the investigated traits, 35 were mapped on the reference map. Additionally, five DArTSeq markers were mapped to Sce assembly 02 and one marker (3602388) to 2R, 110.7 cM in the map of Martis et al. (2013) (Table 5; Table SIII, Fig. S2, ESM). Among eight markers which had effects stable across environments, only two DArTSeq markers—5200240 and 7104304—remained unmapped.

#### CGAM

The number of SNPs associated with investigated traits significant at *P* < 0.01 was 11 (Table SIV, Fig. S1, ESM), with 1 and 10 for R-R and PHS-R, respectively. Six of these associations were identified in the *ScBx1* gene. The polymorphism associated with R-R was found in the gene *ScBx4* (in the first intron). Polymorphisms associated with PHS-R were detected in *ScBx1* (six SNPs: two in the promoter sequence, two in the seventh exon and two in the 3′UTR region), *ScBx2* (three SNPs: two in the first exon and one in the second exon) and *ScBx4* (one SNP in the first intron).

The SNP associated with R-R had an effect stable over all experiments. For PHS-R, 6 out of 10 of the associations were stable across all environments. All associations exhibited a positive effect of the ALT allele (resulting from a given SNP) in comparison to the REF allele (the same as present in line L318). No *ScBx* polymorphism was found to be associated with AMY activity.

SNPs significantly and stably associated with the traits of interest (Table 5) were located in the promoter and 3′UTR regulatory sequences of the *ScBx1* gene, predominantly. No SNP associated with the analysed traits was detected in the *ScBx3* gene.

### Evaluation of Associations

To verify whether the SNPs associated with the investigated traits of DILs are also associated with a given phenotype in the unrelated rye inbreds, KASP analysis was performed. Out of 16 SNPs significantly and stably associated with the investigated traits, four SNP markers were chosen for KASP analysis: ScBx5_1593 associated with R-R, 3602388, ScBx1_1367 and ScBx1_4515 associated with PHS-R. The marker ScBx5_1593 was associated with R-R only at *P* < 0.05, but it was included in the analysis as stable, with a large favourable effect.

All markers, both those associated with R-R and those related to PHS-R, identified the phenotype with correctness higher than 86%. Usually, a slightly higher percentage of mismatches (i.e. other phenotype than expected taking into account the results of association analysis) was noted in the case of the experimental set (Table 6). Only moderate resistance to brown rust (evaluation score under 2.5) associated with ALT GG homozygosity was identified better in the experimental set than in the evaluation set.

**Table 6:** Evaluation of accuracy of selected markers in detecting trait phenotype

Marker	Zygote	Associated trait	EXS		EVS	
			Mean value ± sd of the associated trait	% of mismatches^a^	Mean value ± sd of the associated trait	% of mismatches^a^
3,602,388	REF (G/G)	PHS-R	63.84 ± 17.20	11.11	62.73 ± 3.98	0.00
	ALT (A/A)		19.17 ± 11.79	5.56	11.91 ± 6.22	0.00
	heterozygote (G/A)		72.94 ± 0.00^b^	–	–^c^	–
ScBx1_1367	REF (G/G)	PHS-R	17.15 ± 2.72	0.00	9.61 ± 5.94	0.00
	ALT (A/A)		66.43 ± 14.11	5.26	62.32 ± 3.74	0.00
	heterozygote (G/A)		49.32 ± 34.46	–	97.00 ± 0.00^b^	–
ScBx1_4515	REF (T/T)	PHS-R	19.16 ± 11.80	5.56	11.91 ± 6.22	0.00
	ALT (C/C)		63.00 ± 18.73	13.33	60.05 ± 12.29	3.84
	heterozygote (T/C)		49.32 ± 34.46	–	–^c^	–
ScBx5_1593	REF (C/C)	R-R	3.90 ± 0.36	5.00	3.81 ± 0.64	3.85
	ALT (G/G)		2.49 ± 0.48	7.14	2.04 ± 0.32	7.69
	heterozygote (C/G)		3.12 ± 0.80	–	–^c^	–

*REF* homozygote with the same alleles as present in the reference line L318, *ALT* homozygote with alleles resulting from a given SNP

^a^Estimated only for homozygotes

^b^Only one heterozygote present in EVS

^c^No heterozygotes present in EVS

## Discussion

In this study, 78 SNPs associated with three rye traits—resistance to brown rust and pre-harvest sprouting, and α-amylase activity—were identified; nearly 20% of associations were stable over environments.

### Correlation Between Studied Traits

Out of the three investigated traits, two—R-R and AMY—were found to be weakly correlated in one of the experiments. Although the common mechanisms determining these two traits are unknown, there is published evidence that genes/QTL controlling them are linked. Emebiri et al. (2010) showed that in hexaploid wheat, the gene cluster for rust resistance (*Lr37/Sr38/Y17*) on chromosome 2A is significantly associated with late maturity α-amylase (LMA). It can be assumed that the gibberellins (GA), phytohormones that regulate many common processes throughout the plant life cycle, are the common factor linking these two traits. De Vleesschauwer et al. (2012) reported that treatment of wild-type Nipponbare rice with increasing concentrations of GA3 enhanced resistance to *P. graminicola* in a concentration-dependent manner. Conversely, a decrease of endogenous GA levels using the GA biosynthesis inhibitor uniconazole (Izumi et al. 1984) promoted disease susceptibility. The relationship between gibberellin and α-amylase biosynthesis in rice has been reported by Kaneko et al. (2002), who showed that 3-beta-hydroxylase produced by the *OsGA3ox2* gene is important for induction of expression of the *RAmy1A* gene encoding α-amylase. They concluded that the synthesis of active GA in the epithelium is important for α-amylase expression in the endosperm.

Our results show that in the case of another pair of traits—PHS-R and AMY—there is no correlation. A similar conclusion was drawn following studies on biparental rye populations (Twardowska et al. 2005). Although, as shown earlier, α-amylase activity in rye grain and predisposition to pre-harvest sprouting have many, partially overlapping, QTL, their expression may be regulated by different mechanisms (Masojć and Milczarski 2009; Masojć et al. 2011). The independent genetic control of these traits was also reported for wheat (Mares and Mrva 2014). This information is very important for rye breeders, since in view of our results, AMY and PHS resistance should be considered as independent traits in selection.

### Association Analysis

To identify SNPs associated with three traits of rye—resistance to brown rust and pre-harvest sprouting, and activity of α-amylase—two approaches were selected: GWAM based on sequences of DArTSeq markers and CGAM based on polymorphism in *ScBx* genes. We assumed that this is the most effective tool to generate effective molecular markers for these traits. Its potential results from the likelihood of higher mapping resolution because of the utilization of more recombination events in the germplasm’s developmental history (Abdurakhmonov and Abdukarimov 2008). The success of association mapping depends on many factors, among which, the flowering biology is one of the most important (Abdurakhmonov and Abdukarimov 2008). Rye is an open pollinated species with a majority of heterozygous loci. It may decrease LD and result in low precision of association mapping. To overcome this drawback, it is suggested to use a synthetic population like nested association mapping (NAM) in maize (Yu et al. 2008). The plant materials used in our study consisted of a set of diverse inbred lines, mostly highly homozygous. The choice of such a population is, however, a compromise as, on the one hand, it increased mapping precision, and on the other, inbred lines of rye always suffer from heterozygosity. The association analysis method that was used allows for heterozygosity of genotypes, as it relies on the regression of phenotypic values on genetic predictors computed from the numbers of given alleles for SNPs that can take values 0, 1 or 2 (van Eeuwijk et al. 2010).

The population structure, as described by means of eigenanalysis of marker data, was related to the origin of lines in the case of one of the DArTSeq markers; it was shown to differentiate the groups provided by two breeding companies, but it was not unique, and a number of SNPs provided a similar explanation. The structure resulting from polymorphisms in *ScBx* genes had no obvious relation to breeding groups. The sequences of *Bx* genes were never a target of selection in rye breeding programmes.

### Genome-Wide Association Mapping

For genome-wide association mapping, we decided to use one of the genotyping by sequencing methods, DArTSeq technology, because of its unquestionable competitiveness and efficiency. DArTSeq technology enables selection of the genome fraction corresponding predominantly to gene sequences, which is based on the use of a combination of restriction enzymes *Pst*I/*Taq*I. These enzymes separate low copy sequences (most informative for marker discovery and genotyping) from the repetitive fraction of the genome and use the next-generation sequencing platforms (Sansaloni et al. 2011; Courtois et al. 2013). This technology is therefore positioned in the area of high resolution mapping and detailed genetic dissection of traits. Genotyping by sequencing in plants has been recently effectively employed in marker-trait association analysis for e.g. root traits in rice (Courtois et al. 2013), resistance to *Puccinia hordei* in barley (Dracatos et al. 2014) and complex disease resistance traits in bread wheat (Li et al. 2015). To find significant SNP-trait associations in GWAM, we used a statistical method based on a mixed linear model, in which the SNP effect is modelled by regression of the trait on the number of one of the alleles in the genotype. The analysis is based on data from all environments and takes into account the possibility that the regression coefficient is different in different environments, which is taken as evidence of the “allelic effect × environment interaction”. In case of no interaction, the common allelic effect is estimated. The advantage of the method used is that it allows for formal statistical confirmation of significant interactions, unlike the approaches based on separate data analysis in consecutive environments.

In our study, we identified 67 SNPs in DArT sequences that were linked to observed traits, predominantly associated with PHS-R. The majority of identified trait-SNP relationships were affected by the environment. It is worth emphasizing that the climate conditions in June and July, in two consecutive seasons (2013 and 2014), were notably different. For example, in June 2013, the temperatures were extremely high and relatively stable, whereas in June 2014, significant temperature fluctuations were observed. In Szczecin, in July 2014, there was drought and in Choryń, the rainfall in this month was much higher than average. June and July are critical months for the expression of the analysed traits. Nevertheless, the trait relationship with eight SNPs (12%)—seven for PHS-R and one for AMY—turned out to be stable across all environmental conditions represented by two locations and two consecutive seasons.

More than 50% of SNPs in DArT sequences associated with the investigated traits have been mapped on maps constructed previously (Milczarski et al. 2011, 2016), the majority of them on chromosomes 6R and 7R. Map positions of numerous SNPs related to PHS-R and AMY coincide with QTL detected for these traits by Masojć and Milczarski (2009) and by Masojć et al. (2009) using biparental populations. SNPs associated with R-R have been mapped on chromosome 1R to the same region as genes *Pr3*, *Pr4* and *Pr5* (Roux et al. 2004).

### Candidate Gene Association Mapping

For CGAM, *ScBx* genes, isolated and sequenced recently in our group (Bakera et al. 2015), which control the biosynthesis of BXs, seemed to be perfect candidates. As it was underlined by Zhu et al. (2008), CGAM could be an attractive, rapid and inexpensive approach for identifying markers associated with genes/quantitative trait loci (QTL) for various quantitative traits, provided that the function and sequence of candidate genes are known. BXs have been shown to have various unique properties—from allelopathic to human health-beneficial ones (Makowska et al. 2015). The resistance to fungal pathogens and the activity of α-amylase are listed among the features dependent on BX (for review see Niemeyer 2009). In the present study, 11 environmentally stable SNPs found in *ScBx* genes were associated with either R-R (1 SNP) or PHS-R (10 SNPs). Three SNPs—ScBx1_4515, ScBx4_1583 and ScBx4_1627—were previously shown to be associated with BX content in the above ground parts of plants (the first of the listed markers) and roots (the remaining two markers) (Rakoczy-Trojanowska et al. 2016). Most of the identified SNPs were positioned in the promoters, which could be connected with gene expression. According to Zhu et al. (2008), promoter, intron, exon and 5′/3′-untranslated regions are all reasonable targets for identifying candidate gene SNPs, with non-coding regions expected to have higher levels of nucleotide diversity than coding regions. For example, Liu et al. (2013) showed that the polymorphisms identified in the promoter of the *ZmDREB2.7* gene were associated with drought tolerance among maize varieties. The authors suggested that these polymorphisms may influence gene expression in response to drought stress.

Nearly 80% of SNPs were located in the *ScBx1* and *ScBx2* genes and more than half of them in the promoters. It can be assumed that the expression difference of *ScBx1* and *ScBx2* genes causes differences in levels of BX content and, consequently, the levels of traits potentially dependent on HAs. The *ScBx1* and *ScBx2* genes are thought to be the most crucial genes for the whole BX biosynthesis pathway, as *ScBx1* controls the first step, a branch point in BX biosynthesis (the conversion of indole-3-glycerol phosphate to indole, which is then delivered to the monooxygenation reaction), and *ScBx2* is responsible for the synthesis of the first hydroxamic acid: indolin-2-on (Makowska et al. 2015). A significant positive correlation between the expression level of the *ScBx1* gene and the content of BXs in young rye plants has been shown by Groszyk et al. (2013, 2015). La Hovary (2012) found that BX1 and BX2 transcript levels paralleled the concentrations of BXs.

SNPs stably associated with the traits of interest usually were present in non-coding gene regions (introns of *ScB4* and *ScBx5*, 3′UTRs of *ScBx1*) and 3′ regulatory sequences (promoters of *ScBx1*). These polymorphisms can modify protein properties, at least, or impair enzyme synthesis. Bakera et al. (2015) showed that the changes in BX2 and BX4 proteins (the presence of unique α-helices) resulted from indels present in *ScBx2* and *ScBx4* genes, respectively.

### Evaluation of Identified SNPs

In contrast to the results of GWAM, in the case of CGAM, most (64%) associations were not affected by the environment. Altogether, we identified 15 environmentally stable associations with SNPs (8 in DArT sequences and 7 in *ScBx* genes). All these polymorphisms may probably serve as a template for developing effective molecular markers for MAS. As stated above, the weather in summer, in two consecutive seasons, was considerably different. These circumstances show the potential of the detected SNPs. To test the reliability, we performed a validation experiment by applying the SNPs in an independent set of accessions not included in the association analysis (designated as the evaluation set) using the KASP method. This analytical approach is agreed to be a cost-effective and accurate marker assay, especially for genotyping large segregating populations and in the case of numerous samples with a few SNPs (Saxena et al. 2012; Semagn et al. 2014). There are many publications relating either to the technology itself or to its application in crop improvement programmes, e.g. in maize (Ertiro et al. 2015), wheat (Bernardo et al. 2015) or rice (Liu et al. 2016). However, none of them applies rye.

As expected, KASP turned out to be sufficiently sensitive to differentiate between both types of homozygotes and heterozygotes. The precision as well as relative simplicity, cost-effectiveness and quick analysis make KASP worth recommending as a technology in pre-breeding works. Generally, in the evaluation set, a given phenotype correlated with a given allele. Nevertheless, minor mismatches (at most 13.33% mistakenly identified phenotypes) were observed. They might be caused by insufficiently accurate phenotyping in single cases rather than by the genetic determinants. Nevertheless, the accuracy of genotype-based selection at a level higher than 86% may be of interest to breeders, especially as the evaluation set consisted of lines which were not included in the association mapping and were not related by pedigree with the experimental set of DILs, even when they were bred in the same institution or company. Of course, lines in EVS were not entirely unrelated to DILs, as all plant material used in our study belongs to the same Petkus germplasm pool.

Moreover, the obtained results, especially KASP-evaluated markers for dominantly inherited R-R, can be almost directly implemented in practice, e.g. in marker-assisted selection, because of the structure of the analysed population consisting of inbred lines. Nearly 50% of DILs come from the breeding programmes of two Polish breeding companies. As pointed out by Begum et al. (2015), performing association mapping on a panel of adapted breeding lines rather than on a diversity panel provides an opportunity to apply the results directly to breeding programmes.

## Electronic supplementary material

ESM 1 (DOCX 549 kb)
