# Supplementary material for: Identification of Single Nucleotide Polymorphisms Associated with Brown Rust Resistance, α-Amylase Activity and Pre-harvest Sprouting in Rye (Secale cereale L.)
Source: Plant Mol Biol Report. 2017 Apr 26;35(3):366–78. doi: 10.1007/s11105-017-1030-6 (PMC5443880; doi:10.1007/s11105-017-1030-6)
Supplement: Supplementary file 1 — (DOCX 549 kb) [file 11105_2017_1030_MOESM1_ESM.docx]

**Identification of single nucleotide polymorphisms associated with brown rust resistance, α-amylase activity and pre-harvest sprouting in rye (*Secale cereale* L.)**

**PLANT MOLECULAR BIOLOGY REPORTER**

Monika Rakoczy-Trojanowska^1,2^, Małgorzata Targońska-Karasek^1,2^, Paweł Krajewski^3^, Jan Bocianowski^4^, Małgorzata Schollenberger^1^, Wojciech Wakuliński^1^, Paweł Milczarski^5^, Piotr Masojć^5^, Zofia Banaszak^6^, Katarzyna Banaszak^6^, Waldemar Brukwiński^6^, Wacław Orczyk^7^

^1)^Warsaw University of Life Sciences, Warsaw, Poland; ^2)^Polish Academy of Sciences Botanical Garden - Centre For Biological Diversity Conservation in Powsin Warsaw, Poland, ^3)^Institute of Plant Genetics, Polish Academy of Sciences, Poznań, Poland, ^4)^Poznań University of Life Sciences, Poznań, Poland, ^5)^West Pomeranian University of Technology, Szczecin, Poland, ^6)^Danko Plant Breeders LTD, Kościan, Poland, ^7)^The Plant Breeding And Acclimatization Institute - National Research Institute, Radzików, Poland

e-mail address of the corresponding author:

[monika_rakoczy_trojanowska@sggw.pl](mailto:monika_rakoczy_trojanowska@sggw.pl)

Table SI. *ScBx* gene sequences included in SNP identification

| Gene | Total sequence length including 3′UTR and promoter [bp] | 3′UTR length [bp] | Promoter length [bp] |
| --- | --- | --- | --- |
| *ScBx1* (Acc. No. KF636828.1) | 4935 | 317 | 3000 |
| *ScBx2* (Acc. No. KF620524.1) | 2725 | 75 | 999 |
| *ScBx3* (Acc. No. KF636827.1) | 3358 | 36 | 1495 |
| *ScBx4* (Acc. No. KF636826.1) | 3066 | 166 | 1041 |
| *ScBx5* (Acc. No. KF636825.1) | 3269 | 0 | 950 |

Table SII. Analysis of variance for observed traits

| Trait | F-statistics for fixed effects | | | Variance components (std. error) for random effects | | | Broad sense heritability (%) |
| --- | --- | --- | --- | --- | --- | --- | --- |
|  | Year | Location | Year × Location | Line | Line × Year | Line × Location |  |
| R-R | 21.14*** | 175.03*** | 70.02*** | 0.238 (0.036) | 0.016 (0.010) | 0.045 (0.012) | 78.44 |
| PHS-R | 11.57*** | 61.51*** | 25.16*** | 143.4 (38.0) | 47.0 (15.5) | 169.5 (28.5) | 47.69 |
| AMY | 178.28*** | 13.72*** | 630.87*** | 0.279 (0.071) | 0.067 (0.041) | 0.142 (0.048) | 52.45 |

*** – significant at P < 0.001

Table SIII. Associations between SNP and traits: R-R, PHS-R and AMY found in GWAM with allelic substitution effects* and indication of significant interaction with environment (significant associations selected at P < 0.05 with correction for multiple testing by the Benjamini-Hochberg method; allelic effects refer to the ALT allele with respect to the REF allele.

| Marker ID | SNP position  REF  >ALT** | Frequency of alleles | | R-R | | | | | | | PHS-R | | | | | | | AMY | | | | | | | Sce 02 contig/  map position (chromosome, distance) |
| --- | --- | --- | --- | --- | --- | --- | --- | --- | --- | --- | --- | --- | --- | --- | --- | --- | --- | --- | --- | --- | --- | --- | --- | --- | --- |
|  |  | REF | ALT | -log10(P-value) | q-value | Interaction | Allelic effect for experiment: | | | | -log10(P-value) | q-value | Interaction | Allelic effect for experiment | | | | -log10(P-value) | q-value | Interaction | Allelic effect for experiment | | | |  |
|  |  |  |  |  |  |  | Szczecin 2013 | Szczecin 2014 | Choryn 2013 | Choryn 2014 |  |  |  | Szczecin 2013 | Szczecin 2014 | Choryn 2013 | Choryn 2014 |  |  |  | Szczecin 2013 | Szczecin 2014 | Choryn 2013 | Choryn 2014 |  |
| 3363612 | 17:G>C | 0.64 | 0.36 | 4.82 | 0.035 | yes | -0.32 | -0.18 | -0.25 | -0.15 |  |  |  |  |  |  |  |  |  |  |  |  |  |  | - |
| 3346626 | 10:A>T | 0.56 | 0.44 |  |  |  |  |  |  |  | 10.39 | 0.000 | yes | 1.64 | 6.27 | 13.79 | 10.49 |  |  |  |  |  |  |  | - |
| 3348901 | 45:A>G | 0.49 | 0.51 |  |  |  |  |  |  |  | 3.70 | 0.017 | yes | 0.84 | -4.59 | -8.28 | -4.95 |  |  |  |  |  |  |  | 7RL, 143.1^Mil^ |
| 3358400 | 10:T>G | 0.53 | 0.47 |  |  |  |  |  |  |  | 6.34 | 0.000 | yes | 3.94 | 3.58 | 12.29 | 7.04 |  |  |  |  |  |  |  | - |
| 3358883 | 23:T>C | 0.63 | 0.37 |  |  |  |  |  |  |  | 3.83 | 0.014 | yes | -0.24 | 2.18 | 8.91 | 6.91 |  |  |  |  |  |  |  | - |
| 3361999 | 18:T>A | 0.64 | 0.36 |  |  |  |  |  |  |  | 5.41 | 0.001 | yes | -3.78 | -6.01 | -11.33 | -8.71 |  |  |  |  |  |  |  | 7RL, 145.5 ^Mil^ |
| 3362004 | 29:A>G | 0.59 | 0.41 |  |  |  |  |  |  |  | 3.10 | 0.037 | yes | -2.82 | -2.49 | -9.61 | -6.38 |  |  |  |  |  |  |  | c66221 |
| 3362113 | 50:C>T | 0.64 | 0.36 |  |  |  |  |  |  |  | 3.22 | 0.031 | yes | -1.06 | -2.68 | -10.58 | -2.54 |  |  |  |  |  |  |  | 1RS/L, 93.3 ^Mil^ |
| 3362253 | 67:C>A | 0.37 | 0.63 |  |  |  |  |  |  |  | 6.46 | 0.000 | yes | -0.13 | -1.94 | -12.41 | -6.82 |  |  |  |  |  |  |  | 3RL, 185.8 ^Mil^ |
| 3362338 | 32:A>G | 0.66 | 0.34 |  |  |  |  |  |  |  | 3.59 | 0.019 | yes | -1.17 | -5.27 | -10.54 | -3.94 |  |  |  |  |  |  |  | - |
| 3362368 | 46:G>A | 0.57 | 0.43 |  |  |  |  |  |  |  | 5.29 | 0.001 | yes | 1.30 | 1.50 | -8.86 | -5.98 |  |  |  |  |  |  |  | 5RS, 49.7 ^Mil^ |
| 3362499 | 66:G>A | 0.47 | 0.53 |  |  |  |  |  |  |  | 5.07 | 0.002 | yes | 0.63 | -2.39 | -11.21 | -4.56 |  |  |  |  |  |  |  | 7RS/L, 93.9 |
| 3362695 | 42:T>C | 0.40 | 0.60 |  |  |  |  |  |  |  | 3.57 | 0.019 | yes | -1.77 | -2.66 | -7.90 | -8.15 |  |  |  |  |  |  |  | c4659/1R, 125.8 |
| 3362776 | 15:G>A | 0.35 | 0.65 |  |  |  |  |  |  |  | 8.42 | 0.000 | yes | -1.18 | -5.96 | -13.15 | -9.15 |  |  |  |  |  |  |  | 6RL, 98 |
| 3362915 | 43:G>A | 0.65 | 0.35 |  |  |  |  |  |  |  | 2.94 | 0.045 | yes | 1.32 | -3.36 | -9.55 | -4.63 |  |  |  |  |  |  |  | - |
| 3363110 | 22:T>C | 0.40 | 0.60 |  |  |  |  |  |  |  | 3.21 | 0.031 | yes | -0.42 | -3.13 | -9.59 | -4.72 |  |  |  |  |  |  |  | - |
| 3363874 | 42:T>A | 0.35 | 0.65 |  |  |  |  |  |  |  | 3.57 | 0.019 | yes | -1.40 | 1.68 | 6.16 | 8.13 |  |  |  |  |  |  |  | 7RL, 143.1 ^Mil^ |
| 3364051 | 25:G>A | 0.62 | 0.38 |  |  |  |  |  |  |  | 4.93 | 0.002 | yes | 3.60 | -1.36 | -4.75 | -8.20 |  |  |  |  |  |  |  | 6RS, 35.9 ^Mil^ |
| 3364793 | 7:G>A | 0.39 | 0.61 |  |  |  |  |  |  |  | 4.41 | 0.005 | yes | 1.45 | -4.34 | -9.30 | -5.54 |  |  |  |  |  |  |  | - |
| 3364815 | 38:G>A | 0.34 | 0.66 |  |  |  |  |  |  |  | 4.59 | 0.004 | yes | -2.41 | -2.92 | -12.13 | -6.71 |  |  |  |  |  |  |  | 7RL, 134.5 ^Mil^ |
| 3365062 | 41:T>C | 0.44 | 0.56 |  |  |  |  |  |  |  | 3.65 | 0.018 | yes | -0.74 | 3.29 | 8.94 | 5.44 |  |  |  |  |  |  |  | 7RL, 139.3 ^Mil^ |
| 3365136 | 10:G>C | 0.66 | 0.34 |  |  |  |  |  |  |  | 3.18 | 0.033 | yes | -0.68 | 1.63 | 9.44 | 4.28 |  |  |  |  |  |  |  | c8003 |
| 3365354 | 23:A>G | 0.42 | 0.58 |  |  |  |  |  |  |  | 3.98 | 0.012 | yes | -2.57 | -1.15 | -10.23 | -6.56 |  |  |  |  |  |  |  | 7RL, 161.7 ^Mil^ |
| 3577491 | 21:T>C | 0.42 | 0.58 |  |  |  |  |  |  |  | 3.86 | 0.014 | yes | -0.56 | 0.32 | 7.77 | 6.99 |  |  |  |  |  |  |  | 6RS, 46 ^Mil^ |
| 3577614 | 24:G>C | 0.47 | 0.53 |  |  |  |  |  |  |  | 3.11 | 0.037 | yes | 1.37 | -1.74 | -8.66 | -4.30 |  |  |  |  |  |  |  | 6RS, 61.4 ^Mil^ |
| 3586919 | 12:C>T | 0.51 | 0.49 |  |  |  |  |  |  |  | 3.69 | 0.017 | yes | 1.68 | 2.42 | 9.56 | 5.96 |  |  |  |  |  |  |  | 2RL, 144 ^Mil^ |
| 3591466 | 67:C>A | 0.36 | 0.64 |  |  |  |  |  |  |  | 3.84 | 0.014 | yes | -1.29 | -5.61 | -10.17 | -2.22 |  |  |  |  |  |  |  | - |
| 3595321 | 59:A>G | 0.54 | 0.46 |  |  |  |  |  |  |  | 3.49 | 0.022 | yes | -2.18 | -5.39 | -9.41 | -0.64 |  |  |  |  |  |  |  | 1RS, 83 ^Mil^ |
| 3595498 | 53:C>G | 0.47 | 0.53 |  |  |  |  |  |  |  | 3.45 | 0.022 | no | -3.94 | | | |  |  |  |  |  |  |  | 6RS, 15.9 ^Mil^ |
| 3595841 | 57:G>A | 0.41 | 0.59 |  |  |  |  |  |  |  | 3.61 | 0.019 | yes | 0.00 | 0.96 | 8.58 | 5.47 |  |  |  |  |  |  |  | 5RL, 142 ^Mil^ |
| 3595884 | 26:A>C | 0.33 | 0.67 |  |  |  |  |  |  |  | 2.94 | 0.045 | yes | -1.51 | -2.14 | -8.64 | -5.95 |  |  |  |  |  |  |  | - |
| 3596712 | 22:C>G | 0.38 | 0.63 |  |  |  |  |  |  |  | 2.94 | 0.045 | yes | -0.11 | -5.06 | -6.28 | -7.85 |  |  |  |  |  |  |  | - |
| 3596838 | 36:C>T | 0.33 | 0.67 |  |  |  |  |  |  |  | 4.25 | 0.007 | yes | 1.63 | 1.83 | 10.62 | 5.97 |  |  |  |  |  |  |  | 7RL, 143.1 ^Mil^ |
| 3602049 | 12:A>C | 0.58 | 0.42 |  |  |  |  |  |  |  | 3.95 | 0.012 | yes | 2.49 | 5.00 | 10.67 | 5.73 |  |  |  |  |  |  |  | - |
| 3602388 | 28:G>A | 0.43 | 0.57 |  |  |  |  |  |  |  | 3.35 | 0.026 | no | -2.33 | | | |  |  |  |  |  |  |  | c39213/2R,110.7^Mar^ |
| 3602949 | 56:G>C | 0.68 | 0.32 |  |  |  |  |  |  |  | 3.02 | 0.043 | yes | -1.19 | 3.52 | 7.84 | 5.86 |  |  |  |  |  |  |  | 3RL, 105 ^Mil^ |
| 3730851 | 40:G>C | 0.30 | 0.70 |  |  |  |  |  |  |  | 3.32 | 0.027 | yes | 1.60 | 1.81 | 9.22 | 6.44 |  |  |  |  |  |  |  | 6RL, 231 ^Mil^ |
| 3739003 | 19:A>C | 0.30 | 0.70 |  |  |  |  |  |  |  | 4.81 | 0.003 | yes | 0.72 | -1.15 | -10.29 | -5.79 |  |  |  |  |  |  |  | 3RL/105 ^Mil^ |
| 3743000 | 65:T>C | 0.68 | 0.32 |  |  |  |  |  |  |  | 4.59 | 0.004 | yes | -2.88 | -2.80 | -11.70 | -5.48 |  |  |  |  |  |  |  | 1RL, 104.8 ^Mil^ |
| 3743907 | 5:A>C | 0.44 | 0.56 |  |  |  |  |  |  |  | 2.93 | 0.045 | yes | 1.01 | 2.30 | 9.48 | 3.74 |  |  |  |  |  |  |  | - |
| 3745935 | 12:A>G | 0.53 | 0.47 |  |  |  |  |  |  |  | 3.46 | 0.022 | no | -4.72 | | | |  |  |  |  |  |  |  | 6RS, 35.9 ^Mil^ |
| 3891805 | 19:C>T | 0.54 | 0.46 |  |  |  |  |  |  |  | 3.16 | 0.034 | yes | 0.86 | 2.95 | 9.15 | -1.01 |  |  |  |  |  |  |  | - |
| 3892565 | 31:A>G | 0.35 | 0.65 |  |  |  |  |  |  |  | 6.71 | 0.000 | yes | 2.84 | 5.08 | 12.84 | 8.14 |  |  |  |  |  |  |  | 2RL, 148 ^Mil^ |
| 3896073 | 9:G>A | 0.38 | 0.62 |  |  |  |  |  |  |  | 4.84 | 0.003 | yes | 0.38 | 1.48 | 10.43 | 5.67 |  |  |  |  |  |  |  | - |
| 4489001 | 10:A>G | 0.43 | 0.57 |  |  |  |  |  |  |  | 4.16 | 0.008 | yes | 1.61 | 2.16 | 10.13 | 6.34 |  |  |  |  |  |  |  | - |
| 4490415 | 37:A>C | 0.61 | 0.39 |  |  |  |  |  |  |  | 3.08 | 0.038 | yes | 2.61 | 3.73 | 9.67 | 1.17 |  |  |  |  |  |  |  | - |
| 5200240 | 17:G>C | 0.45 | 0.55 |  |  |  |  |  |  |  | 2.94 | 0.045 | no | 3.88 | | | |  |  |  |  |  |  |  | - |
| 5202901 | 6:A>T | 0.61 | 0.39 |  |  |  |  |  |  |  | 3.30 | 0.027 | yes | 3.08 | 2.31 | 10.24 | 3.59 |  |  |  |  |  |  |  | - |
| 5203824 | 8:G>A | 0.54 | 0.46 |  |  |  |  |  |  |  | 4.87 | 0.003 | yes | -0.29 | -2.51 | -10.11 | -6.97 |  |  |  |  |  |  |  | - |
| 5214445 | 7:C>T | 0.35 | 0.65 |  |  |  |  |  |  |  | 3.44 | 0.022 | yes | 0.38 | -0.39 | -8.90 | -5.75 |  |  |  |  |  |  |  | 5RS, 75 ^Mil^ |
| 5217029 | 16:A>G | 0.37 | 0.63 |  |  |  |  |  |  |  | 2.97 | 0.045 | no | 2.81 | | | |  |  |  |  |  |  |  | 5RL, 160 ^Mil^ |
| 5225085 | 12:T>C | 0.49 | 0.51 |  |  |  |  |  |  |  | 3.55 | 0.019 | yes | -1.51 | -2.30 | -9.51 | -5.35 |  |  |  |  |  |  |  | 2RL, 144 ^Mil^ |
| 5226519 | 7:C>G | 0.42 | 0.58 |  |  |  |  |  |  |  | 3.70 | 0.017 | yes | -1.39 | -1.10 | 8.01 | 5.27 |  |  |  |  |  |  |  | - |
| 5500010 | 7:G>A | 0.33 | 0.67 |  |  |  |  |  |  |  | 3.39 | 0.024 | yes | -2.98 | 1.82 | 7.63 | 5.09 |  |  |  |  |  |  |  | 7RS, 70 ^Mil^ |
| 5500484 | 19:C>T | 0.61 | 0.39 |  |  |  |  |  |  |  | 5.40 | 0.001 | yes | -3.09 | 0.96 | -11.63 | -4.25 |  |  |  |  |  |  |  | - |
| 5797745 | 5:T>A | 0.43 | 0.57 |  |  |  |  |  |  |  | 5.77 | 0.001 | yes | 3.31 | -2.26 | -8.51 | -6.90 |  |  |  |  |  |  |  | 5RL, 160 ^Mil^ |
| 5802575 | 24:T>C | 0.39 | 0.61 |  |  |  |  |  |  |  | 3.23 | 0.031 | no | -3.16 | | | |  |  |  |  |  |  |  | 3RL, 105 |
| 5803969 | 43:T>C | 0.41 | 0.59 |  |  |  |  |  |  |  | 4.77 | 0.003 | yes | 1.74 | 6.39 | 11.06 | 5.47 |  |  |  |  |  |  |  | - |
| 5804179 | 26:G>C | 0.59 | 0.41 |  |  |  |  |  |  |  | 2.96 | 0.045 | yes | 1.13 | -1.48 | -8.98 | -2.47 |  |  |  |  |  |  |  | - |
| 5804636 | 6:A>C | 0.38 | 0.62 |  |  |  |  |  |  |  | 2.92 | 0.045 | yes | -1.94 | -2.61 | -8.28 | -7.11 |  |  |  |  |  |  |  | - |
| 6210975 | 21:C>T | 0.51 | 0.49 |  |  |  |  |  |  |  | 3.31 | 0.027 | yes | -2.49 | -4.91 | -6.66 | 2.97 |  |  |  |  |  |  |  | 2RL, 144 ^Mil^ |
| 7104304 | 19:T>C | 0.43 | 0.57 |  |  |  |  |  |  |  | 2.94 | 0.045 | no | 1.06 | | | |  |  |  |  |  |  |  | - |
| 3364124 | 67:A>C | 0.35 | 0.65 |  |  |  |  |  |  |  |  |  |  |  |  |  |  | 4.06 | 0.044 | yes | -0.16 | -0.03 | -0.29 | -0.44 | c18513 |
| 3364905 | 42:G>T | 0.34 | 0.66 |  |  |  |  |  |  |  |  |  |  |  |  |  |  | 4.33 | 0.036 | yes | -0.10 | 0.15 | -0.15 | -0.34 | 5RL, 170.2 ^Mil^ |
| 3581291 | 67:A>C | 0.41 | 0.59 |  |  |  |  |  |  |  |  |  |  |  |  |  |  | 4.02 | 0.044 | no | 0.24 | | | | 1RL, 168.7 ^Mil^ |
| 3584097 | 45:C>G | 0.69 | 0.31 |  |  |  |  |  |  |  |  |  |  |  |  |  |  | 5.46 | 0.008 | yes | -0.01 | 0.10 | 0.12 | 0.51 | - |
| 3585537 | 24:G>A | 0.46 | 0.54 |  |  |  |  |  |  |  |  |  |  |  |  |  |  | 4.78 | 0.019 | yes | -0.16 | 0.00 | -0.13 | -0.45 | - |

^Mil^ –maps of Milczarski et al.( 2011); Milczarski et al. (2016); Bolibok-Brągoszewska (unpublished) and Milczarski et al. (unpublished).

^Mar^ – map of Martis and al. (2013)

*) mutant with respect to reference - negative value means decrease of the trait level in mutant homozygote)

**) REF – allele present in the reference line L318; ALT - allele resulting from SNP

Table SIV. Associations between SNP and observed traits found in CGAM significant at P < 0.01 with allelic substitution effects and an indication of significant interaction with environment

| SNP ID (gene, position) | Allele REF* | Allele ALT** | Frequency | | R-R | | | | | | PHS-R | | | | | |
| --- | --- | --- | --- | --- | --- | --- | --- | --- | --- | --- | --- | --- | --- | --- | --- | --- |
|  |  |  |  |  | -log10(P-value)* | Interaction | Allelic effect for experiment: | | | | -log10(P-value)* | Interaction | Allelic effect for experiment: | | | |
|  |  |  | Allele REF | Allele ALT |  |  | Szczecin 2013 | Szczecin 2014 | Choryn 2013 | Choryn 2014 |  |  | Szczecin 2013 | Szczecin 2014 | Choryn 2013 | Choryn 2014 |
| ScBx4_1583 | T | C | 0.79 | 0.21 | 2.24 | no | -0.14 | | | |  |  |  |  |  |  |
| ScBx1_1367 | G | A | 0.57 | 0.43 |  |  |  |  |  |  | 2.35 | no | 2.82 | | | |
| ScBx1_2474 | C | A | 0.58 | 0.42 |  |  |  |  |  |  | 2.08 | no | 2.68 | | | |
| ScBx1_4491 | G | A | 0.33 | 0.67 |  |  |  |  |  |  | 2.22 | yes | 4.29 | -2.83 | -13.13 | -9.41 |
| ScBx1_4515 | T | C | 0.57 | 0.43 |  |  |  |  |  |  | 2.19 | no | 2.61 | | | |
| ScBx1_4663 | A | G | 0.58 | 0.42 |  |  |  |  |  |  | 2.53 | no | 2.70 | | | |
| ScBx1_4736 | G | C | 0.58 | 0.42 |  |  |  |  |  |  | 2.20 | no | 2.36 | | | |
| ScBx2_1458 | A | C | 0.63 | 0.37 |  |  |  |  |  |  | 2.08 | yes | -3.26 | 3.05 | 8.85 | 7.87 |
| ScBx2_1462 | C | T | 0.63 | 0.37 |  |  |  |  |  |  | 2.06 | yes | -3.34 | 2.92 | 8.79 | 7.80 |
| ScBx2_2614 | G | A | 0.64 | 0.36 |  |  |  |  |  |  | 2.10 | yes | -3.40 | 1.84 | 9.39 | 7.34 |
| ScBx4_1627 | T | C | 0.21 | 0.79 |  |  |  |  |  |  | 2.17 | no | 2.97 | | | |

*) REF – allele present in the reference line L318

**) ALT - allele with SNP


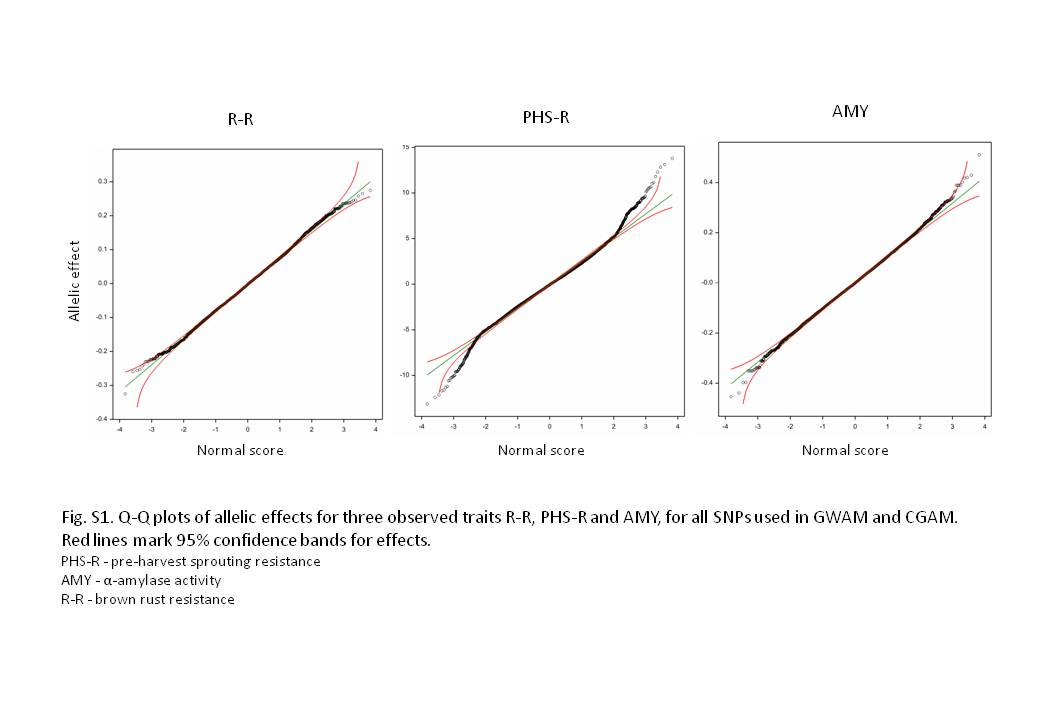


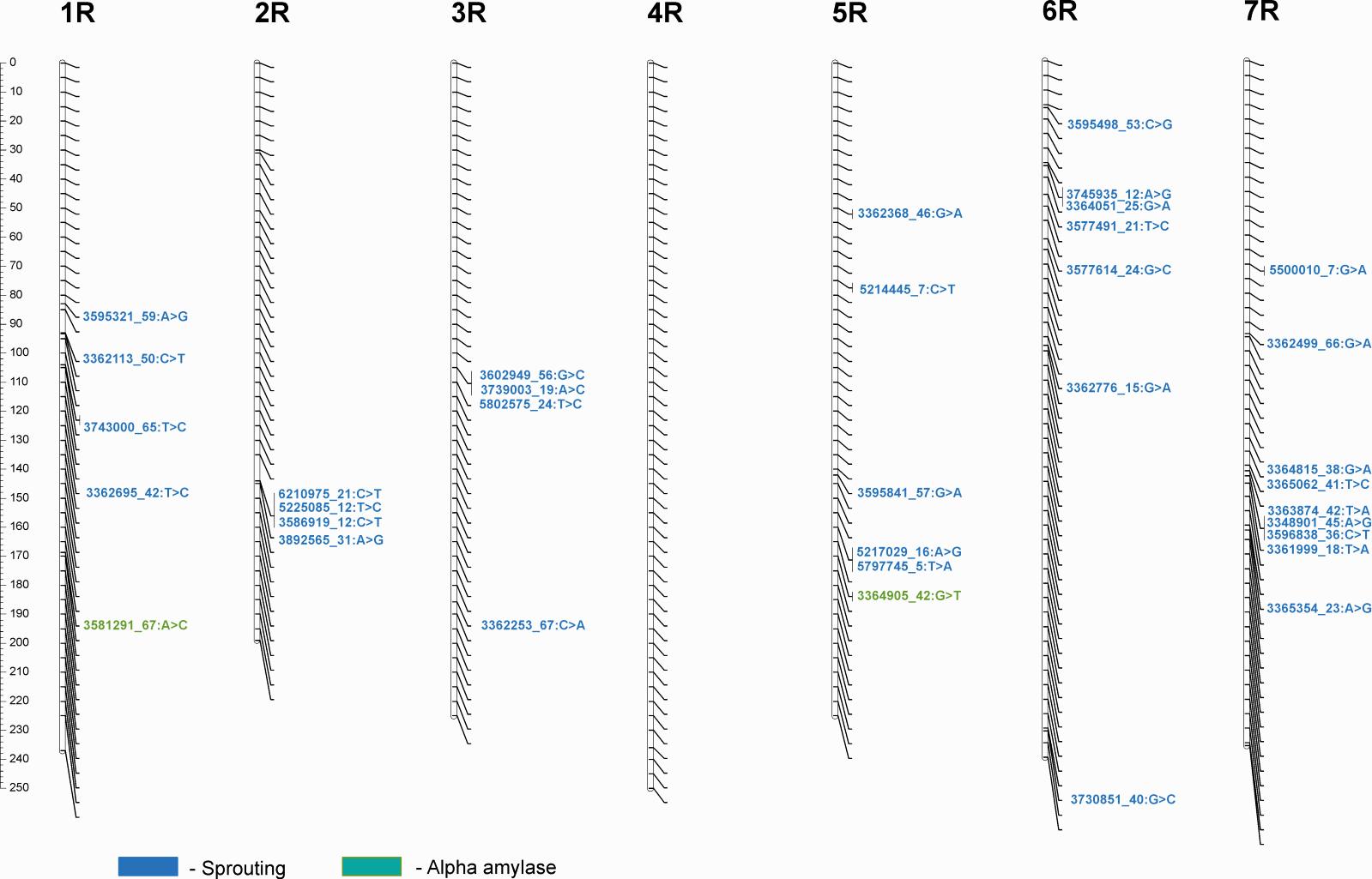


Fig. S.2. Positions of DArTSeq markers identified in GWAM on map based on maps of Milczarski et al.( 2011); Milczarski et al. (2016); Bolibok-Brągoszewska (unpublished) and Milczarski et al. (unpublished).
